# ViennaRNA Package 2.0

**DOI:** 10.1186/1748-7188-6-26

**Published:** 2011-11-24

**Authors:** Ronny Lorenz, Stephan H Bernhart, Christian Höner zu Siederdissen, Hakim Tafer, Christoph Flamm, Peter F Stadler, Ivo L Hofacker

**Affiliations:** 1 Institute for Theoretical Chemistry and Structural Biology, University of Vienna, Währingerstraße 17/3, A-1090 Vienna, Austria; 2 Bioinformatics Group, Department of Computer Science, and Interdisciplinary Center for Bioinformatics, University of Leipzig, Härtelstraße 16-18, D-04107 Leipzig, Germany; 3 Center for non-coding RNA in Technology and Health, University of Copenhagen, Grønnegårdsvej 3, DK-1870 Frederiksberg C, Denmark; 4 Max Planck Institute for Mathematics in the Sciences, Inselstraße 22 D-04103 Leipzig, Germany; 5 Fraunhofer Institute for Cell Therapy and Immunology, Perlickstraße 1, D-04103 Leipzig, Germany; 6 Santa Fe Institute, 1399 Hyde Park Rd, Santa Fe, NM 87501, USA; 7 Research Group Bioinformatics and Computational Biology, Faculty of Computer Science, University of Vienna, Währingerstraße 17/3, A-1090 Vienna, Austria

## Abstract

**Background:**

Secondary structure forms an important intermediate level of description of nucleic acids that encapsulates the dominating part of the folding energy, is often well conserved in evolution, and is routinely used as a basis to explain experimental findings. Based on carefully measured thermodynamic parameters, exact dynamic programming algorithms can be used to compute ground states, base pairing probabilities, as well as thermodynamic properties.

**Results:**

The ViennaRNA Package has been a widely used compilation of RNA secondary structure related computer programs for nearly two decades. Major changes in the structure of the standard energy model, the *Turner 2004 *parameters, the pervasive use of multi-core CPUs, and an increasing number of algorithmic variants prompted a major technical overhaul of both the underlying RNAlib and the interactive user programs. New features include an expanded repertoire of tools to assess RNA-RNA interactions and restricted ensembles of structures, additional output information such as *centroid *structures and *maximum expected accuracy *structures derived from base pairing probabilities, or *z*-*scores *for locally stable secondary structures, and support for input in fasta format. Updates were implemented without compromising the computational efficiency of the core algorithms and ensuring compatibility with earlier versions.

**Conclusions:**

The ViennaRNA Package 2.0, supporting concurrent computations via OpenMP, can be downloaded from http://www.tbi.univie.ac.at/RNA.

## Background

A typical single stranded-nucleic acid molecule has the propensity to form double helical structures causing the molecule to fold back onto itself. Simple rules of complementary base pairing govern this process, which results in a regular pattern of Watson-Crick and GU pairings (helices) and intervening stretches of less regularly ordered nucleotides (loops), collectively known as the molecule's *secondary structure*. Secondary structure elements may be placed in close spatial proximity allowing additional non-covalent interactions. These are not as frequent and often are energetically less favorable compared to canonical base pairs, thus rendering the 3-dimensional tertiary structure of an RNA to be dominated by the underlying scaffold of the secondary structure. The canonical base pairing governs not only the thermodynamics but also the folding kinetics, which can be approximated as a hierarchical process in which secondary structure is formed before tertiary structure [1].

The dominance of base pairing and the confinement to a single interaction partner makes it possible to model RNA (and DNA) secondary structures at a purely combinatorial level, completely ignoring both atom-scale details and spatial embeddings. Formally, an RNA secondary structure is a (labeled) graph whose nodes represent nucleotides. The edge set contains edges between consecutive nodes (*i, i *+ 1) representing the phosphate backbone as well as edges between base pairs. For the latter, the following conditions must hold:

1. base pair edges are formed only between nucleotides that form Watson-Crick or GU base pairs;

2. no two base pair edges emanate from the same vertex, i.e., a secondary structure is a matching;

3. base pair edges span at least three unpaired bases;

4. if the vertices are placed in 5' to 3' order on the circumference of a circle and edges are drawn as straight lines, no two edges cross.

The last condition ensures that the graph is outerplanar and therefore excludes so-called pseudo-knots. Matching problems usually have cost functions determined by edge-weights. The earliest predictions of RNA secondary structures in the early 1970s indeed used such simple energy models [2]. Detailed melting experiments, however, soon showed that a different, more complex type of energy function is necessary to properly model the thermodynamics of nucleic acid structures. Instead of individual base pairs, the energy contributions are dominated by base-pair stacking and the destabilizing entropic effects of unpaired "loops". Sequence-dependent energy parameters for these building blocks contribute to a very good approximation additively to the folding energy [3]. Over the last two decades, this additive standard energy model has been repeatedly refined and updated, see e.g. [4-9].

The RNA folding problem is solvable by means of dynamic programming. The simplest version, known as *maximum circular matching problem*, accounts for base pairing energies only [10,11]. In the early 1980s Nussinov and Jacobson [12] and Michael Zuker with collaborators [13,14] demonstrated that the loop-based energy model is also amenable to the same algorithmic ideas. Their work made computational RNA structure prediction accurate and efficient enough for practical use, resulting in the first versions of mfold. A decade later, John McCaskill realized that the dynamic programming recursions can be adapted to compute the partition function of an equilibrium ensemble of RNA molecules [15], paving the way for efficient computational access to accurate thermodynamic modelling without exceeding an asymptotic time complexity of $O\left(n^{3}\right)$.

The secondary structure model of RNA perfectly fits together with modern genomics and transcriptomics since it works at the same level of abstraction, treating nucleotides as basic entities. With the increasing availability of RNA sequence data, and the realization that many of the functional RNAs have evolutionary well-conserved secondary structures, many research groups developed a plethora of specialized tools for various aspects of RNA bioinformatics. As an alternative to the direct measurement of thermodynamic parameters, for instance, machine learning approaches employing stochastic context free grammars (SCFG) were introduced e.g. in the infernal suite [16,17]. The algorithmic work horses of the SCFG approach, the Cocke-Younger-Kasami (CYK), the inside and the outside algorithms, are also dynamic programming schemes. They are, in fact, very close cousins of the minimum free energy and partition function folding algorithms. The contrafold tools in fact recently bridged the apparent gap between the thermodynamic and the machine learning approach to RNA bioinformatics proposing to learn a parameter set for a SCFG that structurally matches the standard energy model [18].

Several other tools implement dynamic programming based RNA secondary structures prediction: UNAfold [19] is the successor of the original mfold program and adds suppport for predicting RNA-RNA hybridization. RNAstructure [20] started as a reimplementation of mfold with a graphical user interface in Windows, but is now available for other platforms and has added several additional algorithms such as partition function folding and suboptimal structures. The NUPACK suite [21] focuses on folding of several interacting RNA strands and design problems. The group around Kiyoshi Asai developed several tools focusing the usage of centroid and maximum expected accuracy (MEA) estimators, see e.g. [22]. Ye Ding's Sfold program [23] was the first to introduce stochastic structure sampling. The group around Robert Giegerich provides several RNA related tools, notably the RNAshapes [24] program.

The Vienna RNA Package [25] has its roots in a series of large-scale simulation studies aiming at an understanding of adaptive evolution on rugged fitness landscapes [26-28] and the statistical properties of the sequence-structure relationships of RNA [29-31] rather than the detailed analysis of individual RNA molecules of biological interest. The primary design goals for its implementation in the early 1990s, therefore, were twofold. First and foremost, the basic folding algorithms were to be implemented so as to be as efficient as possible in their usage of both CPU and memory resources. The core algorithms are accessible as a C library, which later on was also equipped with Perl bindings to facilitate interoperability with this commonly used scripting language. Secondly, the interactive programs were to be used mostly in (shell-script) pipelines, hence they use a simple command-line interface and, where possible, they read from and write to a stream. This feature made it easy to construct a suite of web services [32] providing easy access to most functionalities of the Vienna RNA Package. With the rising tide of first genomics and then transcriptomics data, the need for both efficient implementation and easy incorporation into pipelines remained, even though the focus gradually shifted from large-scale simulation to large-scale data analysis. Little has changed in the core folding algorithms in the 17 years since the first publication [25] of the package. On the other hand, a variety of variants have been included such as consensus structure prediction from alignments or scanning versions capable of dealing with local structures in genome-scale data sets. The systematic overhaul of the Vienna RNA Package documented here was largely triggered by the publication of improved parametrizations of the energy model, which affected nearly every component in the library, and by the progress in computer technology, which led to the widespread deployment of shared-memory multi-core processors. In order to exploit these hardware features a restructuring of the RNA library to make it thread-safe and hence fit for use in concurrent computations was required. Beyond these technical improvements, the Vienna RNA Package 2.0 features a number of additions to its algorithmic repertoire, an improved API to RNAlib, and an expanded toolkit of auxiliary programs.

### Interactive tools

Since its first release, the ViennaRNA Package included interactive command-line tools which enable users to access the high performance implementations of the algorithms via a command-line interface. To ensure scalability of the use-cases all programs were developed with the objective of handling input- and output-streams, facilitating their integration into *UNIX pipes*. Thus pre- and post-processing of the input/output data can proceed without the need of intermediate input- or output-files. Most programs of the ViennaRNA Package furthermore are able to operate in *batch mode*, handling large sets of input data with a single call. By default, the programs of the ViennaRNA Package generate an output that is meant to be easily parsable while keeping it human-readable.

**Figure 1:**
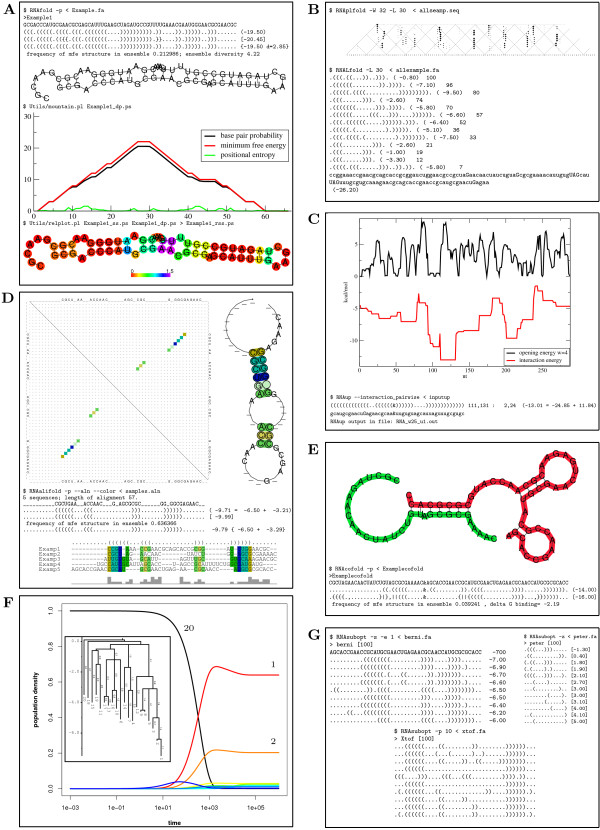
**Example calls of programs included in the **ViennaRNA Package** and their corresponding output**. (**A**) Single sequence analysis using RNAfold. (**B**) Locally optimal secondary structures and base pair probabilities using RNAplfold and RNALfold. (**C**) Interaction thermodynamics of two RNA sequences computed by RNAup. (**D**) Consensus structures and base pair probabilities for RNA sequence alignments obtained from RNAalifold. (E) Secondary structure of an RNA dimer calculated by RNAcofold. (F) Folding kinetics using RNAsubopt in conjunction with the external programs barriers and treekin. (**G**) Suboptimal secondary structures generated by RNAsubopt. For a detailed description see the appendix.

The core of the package provides several variants of the RNA folding recursion: energy minimization, partition function and base pairing probabilities, backtracing of suboptimal structures, alignment-based as well as scanning versions. The decision whether a certain functionality is implemented as a separate stand alone program or as an optional command-line switch is based on the compatibility of I/O formats and internal data structures. Table 1 presents the implemented model variants as well as the data formats for each program, whereas Figure 1 illustrates example program calls together with their corresponding output. In the following paragraphs, we provide a comprehensive summary of programs included in the ViennaRNA Package.

**Table 1:** Main features of the interactive programs provided by the ViennaRNA Package 2.0

Program				Energy model variants							Data formats		
	***intramolecular bp***	***intermolecular bp***	***structure constraint***	***canonical structures***	***circular sequence***	***dangling end model(s)***	***centroid structure***	***MEA structure***	***suboptimal structures***	***base pair probabilities/partition function***	***input format(s)***	***text output file(s)***	***PostScript plot(s)***


		*single sequence analysis (global variant)*											

RNAfold	+	-	+	+	+	0,1,2,3	+	+	-	+	F,V	-	+
RNAsubopt	+	+	+	+	+	0,1,2,3	NA	NA	B,E,Z	-	F,V	-	-
RNAcofold	+	+	+	+	NA	0,1,2,3	-	-	-	+	F,V	+	+
RNAup	+	+	+	+	-	0,2	-	-	-	+	V	+	-
RNAduplex	-	+	-	+	-	0,1,2,3	-	-	E	-	V	+	-
RNA2Dfold*	+	-	-	-	+	0,2	-	+	B	-	V	-	-
RNAPKplex*	+	-	-	+	-	0,1,2,3	-	-	E	-	F,V	-	+
RNAplex*	-	+	+	-	-	2	NA	NA	E	-	V,W	+	+
RNAsnoop*	+	+	+	+	-	2	NA	NA	E	-	V,W	+	+


			*single sequence analysis (local variant)*										

RNALfold	+	-	-	+	-	0,1,2,3	-	-	-	-	F,V	-	-
RNAplfold	+	-	-	+	-	0,2	-	-	-	-	F,V	+	+


			*comparative analysis (global variant)*										

RNAalifold	+	-	+	+	+	0,2	+	+	B	+	C,S	-	+
RNAaliduplex	-	+	-	+	-	0,1,2,3	-	-	E	-	C,S	+	+


			*comparative analysis (local variant)*										

RNALalifold*	+	-	-	+	-	0,1,2,3	-	-	-	+	C,S	+	+


				*Misc. analysis/Utilities*									

RNAeval	+	+	NA	NA	+	0,1,2,3	NA	NA	NA	NA	F,V	-	-
RNAplot	NA	NA	NA	NA	+	NA	NA	NA	NA	NA	F,V	-	+
RNAheat	+	-	-	+	-	0,2	-	-	-	-	F,V	-	-
RNAinverse	+	-	NA	NA	-	0,1,2,3	NA	NA	NA	NA	V	-	-
RNApaln	+	-	-	+	-	0,1,2,3	NA	NA	NA	+	V	+	+
RNApdist	+	-	-	-	-	0,1,2,3	NA	NA	NA	+	V	+	+
RNAdistance	+	-	NA	NA	NA	NA	NA	NA	NA	NA	V	+	+

The characters + and - show presence and absence of a certain feature, while NA indicates that the feature is not applicable in a given context. Abbreviations of input file formats are (**C**)lustal-format, (**F**)asta-format, (**S**)tockholm-format, and (**V**)iennaRNA-format. Support for prediction of suboptimal structures may be implemented as (**B**)oltzmann weighted sampling, exhaustive (**E**)numeration of all structures in a given energy band, and (**Z**)uker-style suboptimal structures. Programs marked by an asterisk (*) were not included in a previous release of the ViennaRNA Package.

### Folding

The main secondary structure prediction tool is RNAfold, which computes the minimum free energy (MFE) and backtraces an optimal secondary structure. Using the -p option, RNAfold also uses McCaskill's algorithm [15] to compute the partition function, the matrix of base pairing probabilities, and the centroid structure. The RNAfold output is a string representation of the structure and the folding energy written to the standard output stream. With the -p option, it also creates a PostScript file containing the base pairing probability matrix. Circular RNA sequences are rare in nature and appear infrequently in practical applications. With the --circ option this case is handled as a post-processing for the forward recursion and a preprocessing of the backward recursions without compromising the performance of the folding algorithms for linear RNAs [33]. Constraints can be supplied to the folding algorithms enforcing that individual positions are paired, unpaired, or paired with specific partners.

The program RNAsubopt can be used to generate suboptimal structures. Using command-line options, it can switch between three different ways of generating them: by default, it generates the complete set of suboptimal structures within a certain energy band, the size of which can be chosen using the -e option [34]. With the -p option it uses stochastic backtracking [35] from the partition function to generate a Boltzmann-weighted random sample of structures, effectively providing the functionality of sfold [23]. Finally, the -z option generates suboptimal secondary structures according to Zuker's algorithm [36]. The resulting set consists, for each basepair (*i, j*) that can be formed by the input structure, of the energetically most favorable structure that contains the (*i, j*)-pair. This option implements a feature that has been used frequently in applications of the mfold package.

RNALfold [37] is a "scanning" version of the folding programs that can be used to calculate local stable substructures of very long RNA molecules. Local in this context means that the sequence interval spanned by a base pair is limited by a user-defined upper bound (set by the -L option). Scanning versions of RNA folding programs conceptutally perform computations for all sequence-windows of a fixed size. Algorithmically, they are faster than the naïve approach by re-using partial results for overlapping windows. RNALfold does not come with a partition function version because the global partition function with restricted base pair span is of limited interest in practical applications. Instead, a separate program, RNAplfold [38], computes the base pairing probability averaged over all sequence windows that contain the putative pair. This tool can also be used to compute the local accessibilities, i.e., the probabilities that sequence intervals are single-stranded in thermodynamic equilibrium (option -a).

RNA2Dfold [39] implements energy minimization, partition function computations, and stochastic backtracing for the two dimensional projection of the secondary structure space that is defined by the base pair distances from the two prescribed reference structures. The restricted ensembles of secondary structures are useful in particular for tracing refolding pathways and to compute lower bounds of energy barriers between alternative conformations of an RNA molecule. Although RNA2Dfold is based upon the usual dynamic programming recursion of energy-directed folding, the asymptotic time complexity is multiplied by a factor of *k*^2 ^· *l*^2^, where *k *and *l *are maximum base pair distances to the first and the second reference structure, resp. Hence, the overall time complexity for a sequence of length *n *is $O\left(n^{7}\right)$. The memory requirements of $O\left(n^{4}\right)$ are also higher than for the regular secondary structure prediction scheme. However, since the implementation uses a sparse matrix approach, the prefactor of time and memory complexity is very small, making the program applicable for RNA sequence lengths of up to about 400 - 600 nt.

### RNA-RNA interactions

Several programs focus on various aspects of the hybridization structure of two RNA molecules, using different levels of detail. The programs RNAcofold [40] and RNAup [41] are two complementary programs with the highest level of detail available within the ViennaRNA Package. RNAup first computes local opening energies for both molecules and then computes interaction energies, looking for the best interaction site of two molecules. RNAcofold, on the other hand, concatenates two molecules and computes a common secondary structure using modified energies for the loop that contains the cut. RNAcofold thus can generate arbitrary many binding sites, but does not allow pseudoknotted configurations, while RNAup covers only a single interaction site, which however may form a complex pseudoknotted configuration. The partition function version of RNAcofold can be used to investigate the concentration dependency of dimerization, similar to [42]. On the other hand, RNAup is mostly geared towards investigations of the binding of regulatory RNA molecules with their target RNAs.

RNAPKplex is at present the only component of the Vienna RNA Package that explicitly predicts pseudoknotted RNA structures [43]. As an "intramolecular variant" of RNAup it computes accessibilities and then identifies regions that can form stable base pairs.

Although optimized for speed, the full-fledged folding algorithms are not fast enough for genome-wide applications. RNAduplex, similar to Rehmsmeier's RNAhybrid [44], ignores intramolecular structures and all multi-branch loops in its search for thermodynamically favorable interaction regions. RNAplex [45] achieves a massive gain in speed by simplifying the energy model for interior loops to an affine gap cost model, effectively reducing the folding problem to a variant of local sequence alignment. The accuracy of this approach can be further improved by reading in accessibilities (as computed by RNAplfold) and incorporating them into the scoring model [46].

The specialized programs RNAsnoop [47] for the prediction of target sites of H/ACA snoRNAs, and RNALfoldz [48] for the evaluation of predicted local secondary structures, use SVMs to further classify the output of the RNA folding routines.

### Consensus structures and alignments

A central issue for the comparative analysis of RNA sequences is the computation of a consensus structure. Starting from a sequence alignment, this can be achieved using the same algorithmic framework as folding a single sequence. More precisely, energy contributions can be added up in a columnwise manner to yield an effective energy model for the alignment as a whole [49]. The Vienna RNA Package provides alignment-based variants for several of the algorithms discussed above: RNAalifold [50] computes global consensus structures both in MFE and partition function mode, a scanning version of long sequence alignments is RNALalifold. RNAaliduplex is designed to facility the search for conserved RNA-RNA interaction sites in large alignment data sets. The alidot program [51,52], finally, extracts local conserved structures given a sequence alignment and secondary structure predictions for each of the aligned sequences. By default, consensus structure prediction is dominated by the thermodynamic parameters and sequence covariation. Thus, phylogenetic support for conservation of secondary structure is included only as a small bonus energy term. A much more sophisticated substitution model for paired regions based on the RIBOSUM scoring scheme [53] can be invoked with the -R option.

The Vienna RNA Package does not contain its own optimized implementation for the *simultaneous *folding and alignment of two RNA sequences, i.e., of the Sankoff algorithm [54]. We refer to the well-established software tools FoldAlign [55], or DynAlign [56] for this task. A simplified version of the Sankoff algorithm underlies pmcomp [57,58], a facility to align pre-computed base-pairing probability matrices, although this tool is now included mostly for backward compatibility. An improved and much more efficient implementation is provided by the locarna package [59] developed in cooperation with Rolf Backofen and Sebastian Will and distributed separately.

With RNApaln and RNApdist the package also provides tools to align and compare base pair probability patterns using modified string alignment algorithms. Tree editing distances and corresponding pairwise alignments can be computed with RNAdist.

### Miscellaneous tools

Concerning sequence design, we ship the program RNAinverse [25]. It generates a sequence that folds into the input structure by mutating a start sequence. More efficient versions of inverse folding algorithms have become available over the last decade, see e.g. INFO-RNA [60], RNA Designer [61] and the recent NUPACK design algorithms [62]. Nevertheless, RNAinverse remains useful for some applications as it is designed for search for solutions as close as possible to the starting sequence. RNAswitch [63] takes a pair of secondary structures as input and finds a sequence that has both input structures as near ground states. The possibility to design bistable RNAs may be useful e.g. for synthetic biology.

A closer look at the dynamics of RNA folding a available through kinfold [64], a rejectionless Monte Carlo simulation algorithm generating trajectories of subsequent secondary structures. Kinetic information can also be obtained from the exhaustive enumeration of suboptimal structures using RNAsubopt in conjunction with the barriers package [64,65]. The latter is not restricted to RNA landscapes and hence distributed separately from the Vienna RNA Package.

### Auxiliary Programs

In addition to the prediction and analysis tools, the ViennaRNA Package provides utility programs and scripts that mainly assist in processing input- and output data. RNAeval computes the energy of a given structure formed by a given sequence and can in particular be used to re-compute energies for a given pair of sequence and structure with different energy models. The Perl script refold.pl generates single structure predictions using a previously computed consensus structure as constraint.

RNAplot can be used to generate a graphical representation of the an input sequence/structure pair [66]. Several Perl scripts can be used to further manipulate PostScript output produced by the various components of the Vienna RNA Package. Conventional structure drawings can be rotated with rotate_ss.pl. The relplot.pl script includes reliability annotation into secondary structure plots, colorrna.pl uses the conservation of alignments for coloring consensus structure plots, while coloraln.pl does the same with an alignment. Mountain plots can be produced with mountain.pl and cmount.pl from single and consensus structures, respectively.

Many tools in RNA bioinformatics use mfold's "connectivity" (.ct) file format. The dot-bracket representation used consistently by the Vienna RNA Package can converted into this format using b2ct and ct2b.pl, resp.

### The ViennaRNA Webserver

The ViennaRNA Webserver [32] facilitates an easy to use form based web browser interface to most of the programs included in the ViennaRNA Package and additional tools. It combines the call of the appropriate command-line tools with post-processing steps to obtain a visualization of the output. The webtools echo the command-lines used to call components of the Vienna RNA Package; this feature can be used to get more familiar with the individual tools. The webserver also provides an interface to the barriers and treekin program allowing the analysis of folding landscapes and structural refolding kinetics. The backbone of the ViennaRNA Webserver has been upgraded so that all calculations with the webserver profit from the increased performance of the new ViennaRNA Package.

### Modifying the energy parameters of the model

The energy model implemented in ViennaRNA Package 2.0 follows the structure of the *Turner 2004 *energy parameters as described in [9] with a few very minor deviations. Compared to previous parametrizations, the *Turner 2004 *model introduced additional look-up tables for certain free energies and for loop entropies in response to more precise measurements of certain loop types. For the sake of computational efficiency a few peculiar rules were deliberately ignored, however. Details on these discrepancies, which do not affect the overall accuracy of predictions (see below), are provided in the appendix.

All programs of the ViennaRNA Package can read in energy parameters from a human-readable text file allowing the user to replace the default *Turner 2004 *parameter set. This can either be a user-supplied parameter file or one of several parameter compilations that are shipped with the package. Of particular interest are parameters for DNA folding. Here we provide a parameter set compiled by Douglas Turner and David Mathews [67] from published data, incorporating in particular earlier work by the group of John SantaLucia [68]. While the Turner parameters are based almost exclusively on thermodynamic measurements, there has been increasing interest in optimizing parameters such as to maximize prediction accuracy, see e.g. [69]. As an example for such trained parameters we provide the *Andronescu *parameter set from ref. [70].

To maintain backward compatibility we also ship *Turner '99 *energy parameter files containing the basic contributions used in previous versions of the ViennaRNA Package. These parameter files, however, will not always produce results identical to earlier versions of the package. Affecting mainly the computation of consensus structures, these differences are mainly owed to a different handling of non-standard base pairs (i.e., base pairs other than Watson-Crick and GU). The current implementation assumes that the energy contribution of a loop with non-standard base pairs or non-standard nucleotides equals the least stabilizing contribution from the same loop type with canonical nucleotides and pairs only. Small differences may also appear in partition function computations as a consequence of round-off errors.

Since the structure of the energy model has changed in ViennaRNA Package 2.0, energy parameter files for versions 1.8.5. and earlier will not work with the new version of the package. Such old-style user-supplied parameter files can be converted to the new file format using the RNAparconv utility.

#### Additional output options

More information gathered through the course of the folding algorithms can be included in the output. RNAfold and RNAalifold, for instance, optionally provide further information about the reliability of folding results. When evaluating ensemble properties with the partition function, most programs now also compute the *centroid *structure [71], i.e., the structure with the smallest average base pair distance to all other structures in the ensemble. When base pair probabilities are computed, the maximum expected accuracy (MEA) structure [18,72] is also available. The RNALfold/RNALfoldz program now features an add-on to calculate the *z-score *for the predicted local secondary structures [48]. This makes results comparable between sequences with different nucleotide compositions and facilitates the choice of a reasonable cutoff thresholds to decrease the number of structure hits.

### Program options and documentation

Each of the command-line tools provides the option -h or --help to print a brief overview of its general behavior as well as a list of all available parameter options including their description. To obtain more detailed information or even exemplary use-case scenarios for a certain program of the ViennaRNA Package, a *UNIX manpage *is provided for each of them.

An important change in the new release is the compliance to the GNU standard regarding the format of command-line options. Short options consist of a single character preceded by a minus sign, e.g. -p, while long options are strings of two or more characters preceded by two minus signs, e.g. --noLP. This change will break backward compatibility wherever command-line tools from older versions of the package were used. This can be easily fixed by inserting the second dash in long options.

### Input file formats

A plethora of different file formats have been introduced by the many tools and databases relevant to RNA bioinformatics. The ViennaRNA Package has also contributed to this unpleasant diversity with its own native formats. Originally designed for simulation pipelines in which no meta-data is attached to sequence or structure data, it expects input items (sequences and/or structures) as single strings uninterrupted by white spaces or line breaks. FASTA-like headers can optionally be used to specify an identifier for the data item(s). Secondary structures are also specified as strings, using the three characters (, ), and. to denote nucleotides that are paired with a partner upstream or downstream, or that are unpaired, resp. In addition to uniquely determining a pseudoknot-free secondary structure, this notation has the advantage of providing a compact annotation of the sequence or alignment to which the structure refers. The dot-parentheses-format is meanwhile used also in many unrelated tools e.g. [18,21,61,73-79]. Similar annotation strings are used to specify constraints as input to folding algorithms.

The requirement to write input items on a single line usually requires data format conversions for the interactions with most other bioinformatics tools. These usually read and write *FASTA *format [80], which allows white spaces and line breaks arbitrarily interspersed within a sequence. An improved handling of data input now provides full FASTA support for all tools that require only sequences or sequence alignments as input. This should considerably facilitate the use of the ViennaRNA Package. More complex input structures are still required for the tools that compute RNA-RNA interactions, in particular RNAup and RNAcofold.

Programs that process alignment data used clustal format [81] in previous versions of the package. Due to the wide-spread use of the STOCKHOLM format in RNA bioinformatics, e.g. in the Rfam - RNA family database [82]), support for *.stk files has been added.

There are currently no plans to include support for input formats that use heavy markup such as Genbank [83] files or XML-based formats such as BioXSD [84] or RNAML [85].

## RNAlib -

## API to fast and reliable algorithms

The algorithms implemented in the ViennaRNA Package are not only accessible by means of the interactive programs outlined in the previous section but also directly in the form of a C/C++ library. This makes them readily available for third-party programs and, with the help of included Perl-interface, to elaborate scripting pipelines.

### OpenMP thread-safe C/C++ API

Multi-core CPUs have become standard components in off-the-shelf PC hardware. In order to allow the ViennaRNA Package to make use of this increase of computational power, several changes had to be introduced into the API functions of the RNAlib. Although it is possible to parallelize the core folding algorithms [86,87] this requires substantial overheads so that the gain is small unless massively parallel architectures are used. On the other hand, computationally demanding applications of RNA folding typically require the processing of large numbers of input sequences, a task that trivially can be parallelized. The only requirement for enabling concurrent computation on shared memory multi-core systems using OpenMP [88] is that the core algorithms are independent of shared global variables and thus thread-safe. In particular the variables referring to the energy parameters are now deprecated and replaced by additional functions or parameters which have to be passed to functions. A few remaining global variables, which are inaccessible through RNAlib, were made *thread-private *using OpenMP, allowing simultaneous function calls to operate on private copies of these variables. Using the OpenMP framework, third party applications are therefore now able to call RNAlib interfaces, such as MFE or partition function algorithms, in parallel. Limitations concerning the use of different energy models used in concurrent computations are described in detail in the API reference manual. For backward compatibility, the old functions of the previous API remain included in RNAlib but are marked as deprecated. Thus, programs which were developed for binding against the previous versions of RNAlib up to 1.8.5 are still working without limitations when linked against the new library.

### The reference manual

Documentation is an important issue for the usability of the RNAlib API. In previous versions of the ViennaRNA Package, this was addressed by maintaining, in addition to the source code, a *texinfo*-based reference manual containing introductions into the particular problem sets and describing the related library functions. In order to keep this documentation up to date and to decrease the developers' effort in maintaining the manual, we opted to use *in-source *documentation that (a) helps developers who interact with the source code directly and (b) enable to use the doxygen documentation program to generate a comprehensive and always up-to-date reference manual automatically. An HTML and a PDF version are included in the package.

### PERL bindings

Scripting language bindings to the C functions in the RNAlib are made using the SWIG interface compiler. With the ViennaRNA Package, we include bindings for the most important library functions made accessible for the script language Perl. This allows a very easy access to e.g. the folding functions and thus a rapid design of functional pipelines or small programs that exploit the potential of the ViennaRNA Package. Using the SWIG environment bindings for other (scripting) languages including Python and JAVA can be implemented quite easily.

## Performance

We assess the performance of the ViennaRNA Package 2.0 both in terms of computational efficiency and in terms of prediction accuracy. We emphasize that it is not the purpose of this contribution to compare thermodynamics-based prediction algorithms against other approaches to RNA structure prediction. For such a benchmark we refer to the literature, e.g. [18,89,90].

**Figure 2:**
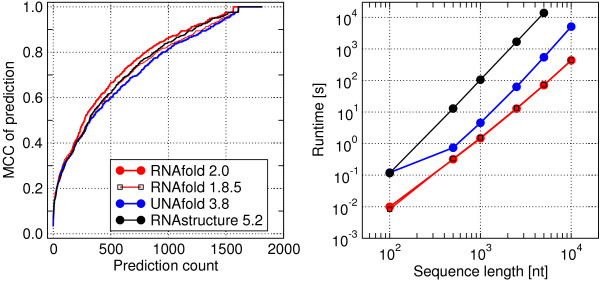
**Performance comparison of RNAfold 2.0 to other secondary structure prediction software**. (A) Accuracy of thermodynamic folding programs in terms of cumulative distribution of the Matthews correlation coefficient (MCC). RNAfold 2.0 outperforms the other secondary structure prediction programs on the RNAstrand dataset: more of its predictions fall into the region of higher performance values. Both versions of RNAfold were run with -d2 option. For UNAFold and RNAStructure default options were used. Performance distributions of Sensitivity, Positive predictive value (PPV) and F-measure are shown in Additional File 1. The averaged overall accuracies can be taken from table 2. (**B**) Comparison of runtimes for MFE structure predictions. Measurement was performed on an Intel^® ^Core™ 2 6600 CPU running at 2.4 GHz. Shown are averaged running times for random sequences of lengths 100 nt (100 samples), 500 nt (100 samples), 1000 nt (100 samples), 2500 nt (20 samples), 5000 nt (16 samples) and 10000 nt (16 samples). While the compared programs RNAfold 2.0, RNAfold 1.8.5 and UNAfold 3.8 were capable of predicting an MFE structure for all tested samples in a relatively small time frame, RNAstructure 5.2 was omitted from predictions for the 10000 nt sample set due to its time requirements.

In order to investigate the impact of the energy parameters, and in particular of our small changes to the *Turner 2004 *model, we use a test set comprising all 1817 non-multimer sequence/structure pairs in the RNAstrand database [73] without pseudoknots in the reference structure. For each sequence, the MFE secondary structure was calculated with RNAfold 2.0, RNAfold 1.8.5, UNAFold 3.8 [19], and RNAstructure 5.2 [20]. All use a nearest neighbor energy model and a variant of Zuker's dynamic programming algorithm. As expected, the new version of RNAfold performs better than the old one. Somewhat surprisingly, however, RNAfold 2.0 also performs slightly better than RNAstructure 5.2 and UNAFold 3.8, despite the fact that we neglected a few peculiarities of the most recent energy model, see Figure 2, Additional File 1 and the implementation details in the appendix. The average performance indicators are compiled in Table 2. We emphasize, however, that the performance of the algorithms differs widely across RNA families and no single implementation provides consistently superior results. Detailed data can be found in Additional File 2.

**Table 2:** Averaged performance measures for thermodynamic folding algorithms

	Sensitivity	Specificity	MCC	F-measure
RNAfold 2.0	0.739	0.792	0.763	0.761
RNAfold 1.8.5	0.711	0.773	0.740	0.737
UNAFold	0.692	0.766	0.727	0.724
RNAStructure	0.715	0.781	0.745	0.742

Despite the increase in the number of parameters from *Turner '99 *to *Turner 2004 *we observe virtually no difference in the runtime and memory consumption between RNAfold 1.8.5 and RNAfold 2.0. Similar comparisons can be made for other components of the ViennaRNA Package. The computational speed of RNAfold compares quite favorably to that of the competing implementations, Figure 2B, although all the implementations of thermodynamic folding algorithms use essentially the same energy model and algorithmic framework, and hence have the same asymptotic runtime and memory consumption.

## Discussion

The ViennaRNA Package has been a useful tool for the RNA bioinformatics community for almost two decades. Quite a few widely-used software tools and data analysis pipelines have been built upon this foundation, either incorporating calls to the interactive programs or directly interfacing to RNAlib. Numeric characteristics of secondary structures, such as Gibbs free energy Δ*G*, Minimum free energy (MFE), ensemble diversity or probabilities of MFE structures in the ensemble, have been widely used as features for machine learning classification, e.g. in microRNA precursor and target detection [91-94]. The non-coding RNA gene finder RNAz [95,96], the snoRNA detector snoReport [97], and RNAstrand [98], a tool that predicts the reading direction of structured RNAs from a multiple sequence alignment, combine thermodynamic properties computed with RNAlib functions and a machine learning component. RNAsoup [99] takes advantage of the programs RNAfold, RNAalifold and some other tools provided by the ViennaRNA Package for a structural clustering of ncRNAs. The siRNA design program RNAxs [100] employs the site accessibility predictions offered by RNAplfold, as does IntaRNA [60], a program to predict RNA interaction sites. Several secondary structure prediction tools, such as CentroidFold [22], McCaskill-MEA [101], or RNAsalsa [102], use base pair probabilities predicted by RNAfold -p as input, while the LocARNA package [59] uses them for structural alignment. The motif-based comparison and alignment tool ExpaRNA [103] and the tree alignment program RNAforester [75] also rely on the algorithms provided by RNAlib. Since its initial publication [25], no comprehensive description [104] of the ViennaRNA Package has appeared. Release 2.0 now implements the latest energy model, provides many new and improved functionalities, and - as we hope - is even easier and more efficient to use due to a thread-safe architecture, an improved API, a more consistent set of options, and a much more detailed documentation. Care has been taken to ensure backward compatibility so that ViennaRNA Package 2.0 can be readily substituted for earlier versions.

## Availability and Requirements

The source code of the ViennaRNA Package as well as the current reference manual can be downloaded from http://www.tbi.univie.ac.at/RNA.

## Competing interests

The authors declare that they have no competing interests.

## Authors' contributions

Work on the Vienna RNA Package is coordinated by ILH. The design and structure of version 2.0 resulted from discussion of RL with ILH, PFS, CF, SHB and CHzS. Implementation and performance analysis was performed by RL with contributions of HT (RNAplex and RNAsnoop). CHzS provided the converted new energy parameter files. Detailed documentation for the RNAlib was done by RL and SHB based on pre-existing sources. The manuscript was written by RL with major contribution by SHB, PFS, and ILH. All authors read and approved the final manuscript.

## Appendix

### Energy model implementation details

The most important technical innovation is the use of the **2004 - improved nearest neighbor model **by Mathews et al. [9] as the default parameter set in all free energy calculations. This entails not only an update of all free energy evaluating sections in each affected program, but also major changes in the structure of the parameter sets. In particular, several additional energy parameters for the different loop types (hairpin loops, interior loops and multi-branch loops) were introduced.

In order to keep the number of energy parameters and thus the complexity of the energy model small, we refrained from implementing exceptional contributions for some highly specialized configurations. In particular the following special cases are not incorporated in our folding recursions:

1. *All-C *loop penalty, i.e., a penalizing contribution for loops consisting of unpaired cytosine only;

2. Additional stabilizing *GU-closure term *that is applied only in the context of hairpin loops, enclosed by a *GU *(not *UG*) base pair which is preceded by two *G*s;

3. A special intramolecular helix formation of the four consecutive base pairs *GC, GU, UG *and *CG*, which has a single tabulated contribution of -4.12 kcal/mol.

4. Consideration of an auxilary contributing factor that reflects the *number of states of a bulge loop*, i.e. the number of all possible bulges with identical sequence.

5. *Average asymmetry *correcting penalty *in multi-branch loops *which constitutes the mean difference in unpaired nucleotides on both sides of the branching stems;

6. Extra penalty for *three-way branching loops with less then two unpaired nucleotides*;

Adapting the dynamic programming recursions to also take into account these loop configurations resulted in an increase of time and memory requirements without a compensating benefit in terms of prediction accuracy. The data-set we used for measuring the prediction performance also did not reveal any significant unfavorable effect of our simplification of the model. However, free energy evaluation of a given sequence/structure pair, as done by RNAeval, may introduce these extra cases in the near future as an additional parameter, such as logarithmic multi-branch loop evaluation.

All our folding algorithms assume -d2 as the default dangling-end model, allowing a single nucleotide to contribute with all its possible favorable interactions. The dangling-end/helix-stacking model suggested by the Turner'04 parameters is realized with the -d3 option. An additional model allowing a single nucleotide to be involved in at most one favorable interaction but ignoring helix-stacking can be chosen with -d1, while -d0 deactivates dangling-end and helix-stacking contributions altogether.

### Performance

The base pair positions along the RNA sequence were taken as predicted properties for all of the performance measurements. Thus, all base pairs in the reference structure contribute to the number of *true positives *(TP). The number of *false positives *(FP) is obtained by counting all base pairs that are in the predicted but not in the reference secondary structure. Along with that, all base pairs present in the reference but not in the prediction result are regarded as *false negatives *(FN). These numbers are then used to compute the *sensitivity*, also known as *true positive rate *(TPR), and *precision*, also known as *positive predictive value *(PPV) [105].

$$\begin{array}{c} \text{TPR}=\frac{\text{TP}}{\text{TP}+\text{FN}}\\ \text{PPV}=\frac{\text{TP}}{\text{TP}+\text{FP}} \end{array}$$

To combine these performance measures into one single value, we used the Matthews Correlation Coefficient (MCC) [106] and the F_1_-score (F-measure), i.e. the harmonic mean of *precision *and *true positive rate.*

$$\begin{array}{c} \text{MCC}=\frac{\text{TP}\cdot \text{TN}-\text{FP}\cdot \text{FN}}{\sqrt{\left(\text{TP}+\text{FP}\right)\left(\text{TP}+\text{FN}\right)\left(\text{TN}+\text{FP}\right)\left(\text{TN}+\text{FN}\right)}}\\ {\text{F}}_{1}=2\cdot \frac{\text{PPV}\cdot \text{TPR}}{\text{PPV}+\text{TPR}} \end{array}$$

Since the total number of possible base pairings is bound by $\frac{1}{2}\cdot n\cdot \left(n-1\right)$, with sequence length *n*, we estimated the number of *true negative *(TN) which is required for calculating the MCC by its upper bound of $\text{TN}=\frac{1}{2}\cdot n\cdot \left(n-1\right)-\text{TP}$.

### Detailed description of Figure 1

Example calls of programs included in the ViennaRNA Package and their corresponding output. (**A**) RNAfold output on a small example sequence. Top: On-screen output - mfe, ensemble representation, and centroid structure as dot-parenthesis (Vienna) representations. Numbers in brackets denote the energies, and the centroid's mean distance to the ensemble. Below: postscript output as generated by the above programm call. The mountain plot and the generating program call are in the center of the sub figure. The bottom shows positional entropy derived reliabilty information color coded into the secondary structure drawing.

**(B) **Example output of programs for local folding. Top: Dot plot as generated by RNAplfold. The plot is a cut out along the diagonal of a quadratic dot plot (see e.g. part **(D) **of this figure). At the bottom, an example output of RNALfold is shown. Local optimal substructures are shown in dot-parenthesis notation together with their energy and the index of their first base.

**(C) **Example output of RNAup. At the bottom the best interacting site between the two input molecules is shown. The xmgrace generated picture above shows the energy necessary to open a window of 4 consecutive bases and the interaction energy that can be achieved when the probe molecule is bound to the target molecule in black and red, respectively.

**(D) **RNAalifold output. At the top and bottom pictures generated by RNAalifold are shown. The conservation of the base pairs is encoded in a color scheme. Red means only one of the 6 possible base pairs is present, ochre means two, green 3 and so on. Paler colors indicate that some of the sequences cannot form a base pair at the respective position in the alignment. The top right corner shows a dot plot. Every dot symbolizes a base pair, the size of the dots at the upper right triangle is proportional to the respective base pair probabilities, while on the lower left triangle the mfe structure is depicted. On the top right the conservation annotated consensus structure drawing can be seen, while on the bottom the annotated alignment is shown. The center of the subfigure shows the on-screen output of RNAalifold. As in the ordinary fold case, the minimum free energy structure, a representation of the ensemble structure and the centroid structure are shown. The energies are split into a thermodynamic part (first) and the conservation part, which are summed to give the total predicted score.

**(E) **RNAcofold output. At the top the secondary structure drawing of the minimum free energy folding of the two molecules is shown. The molecules are color coded to make it easier to tell them apart. The "&" character in the on-screen output below is the separator between the two sequences. In addition to the mfe and the ensemble representation with their energies, the binding energy is shown.

**(F) **Output for kinetics (using RNAsubopt output fed into the external programs barriers and treekin). The diagram shows the change in population from the start, where state 20 is populated, towards the equilibrium state 1. The inner picture shows the barrier tree upon which the relative concentrations of the diagram are based. The 20 lowest suboptimal structures and the paths connecting them are depicted, together with the barrier heights.

**(G) **Output of the three versions of RNAsubopt. Left: Output of the Wuchty algorithm, all structures within a certain energy band are shown. Right: Zuker algorithm, showing the best structures for every possible base pair. Bottom: Stochastic backtracking, random structures drawn according to their probability in the ensemble.

## Supplementary Material

Additional file 1 **Performance comparison (Sensitivity, PPV, F-measure)**. Click here for file

Additional file 2 **Detailed performance comparison**. Click here for file
